# Detecting and identifying the reasons for deleted tweets before they are posted

**DOI:** 10.3389/frai.2023.1219767

**Published:** 2023-09-29

**Authors:** Hamdy Mubarak, Samir Abdaljalil, Azza Nassar, Firoj Alam

**Affiliations:** ^1^Qatar Computing Research Institute, Hamad Bin Khalifa University, Doha, Qatar; ^2^College of Humanities and Social Sciences, Hamad Bin Khalifa University, Doha, Qatar

**Keywords:** disinformation, deleted tweets, hate-speech, Arabic, social media

## Abstract

Social media platforms empower us in several ways, from information dissemination to consumption. While these platforms are useful in promoting citizen journalism, public awareness, etc., they have misuse potential. Malicious users use them to disseminate hate speech, offensive content, rumor, etc. to promote social and political agendas or to harm individuals, entities, and organizations. Oftentimes, general users unconsciously share information without verifying it or unintentionally post harmful messages. Some of such content often gets deleted either by the platform due to the violation of terms and policies or by users themselves for different reasons, e.g., regret. There is a wide range of studies in characterizing, understanding, and predicting deleted content. However, studies that aim to identify the fine-grained reasons (e.g., posts are offensive, hate speech, or no identifiable reason) behind deleted content are limited. In this study, we address an existing gap by identifying and categorizing deleted tweets, especially within the Arabic context. We label them based on fine-grained disinformation categories. We have curated a dataset of 40K tweets, annotated with both coarse and fine-grained labels. Following this, we designed models to predict the likelihood of tweets being deleted and to identify the potential reasons for their deletion. Our experiments, conducted using a variety of classic and transformer models, indicate that performance surpasses the majority baseline (e.g., 25% absolute improvement for fine-grained labels). We believe that such models can assist in moderating social media posts even before they are published.

## 1. Introduction

In the last decade, social media has emerged as a predominant channel for freely sharing content online. Interactions on social media platforms facilitate public discussions on topics ranging from local issues to politics. Feelings of intolerance on these platforms can give rise to and propagate hate speech and offensive content through various communication channels. Such content can exacerbate tensions between different groups, potentially leading to violence among their members. Malicious users, both intentionally and unintentionally, exploit media platforms to influence people's thoughts, disseminate hate speech, sway public opinions, attack the human subconscious, spread offensive content, and fabricate truths, among other actions.

The misuse of social media platforms has turned them into potential grounds for sharing inappropriate posts, misinformation, and disinformation (Zhou et al., 2016; Alam et al., 2022). One type of inappropriate posts is **regrettable posts**. These are posts that contain content that may induce guilt in the author or harm the intended audience (Zhou et al., 2016; Diaz Ferreyra et al., 2023). To further clarify these terms, **misinformation** is defined as “*unintentional mistakes such as inaccurate photo captions, dates, statistics, translations, or taking satire seriously*”. **Disinformation** is “*a fabricated or deliberately manipulated text/speech/visual context and intentionally created conspiracy theories or rumors*”, while **melinformation** is “*defined as true information deliberately shared to cause harm*” (Ireton and Posetti, 2018; Alam et al., 2022).

Such posts often get deleted for various reasons: *(i)* users themselves delete the posts, *(ii)* social media platforms delete them due to breach of community guidelines (Almuhimedi et al., 2013; Chowdhury et al., 2020). Sleeper et al. (2013) examined regrets within in-person and virtual conversations. They found that Twitter users tend to delete tweets or sometimes apologize once they realize their regret. The potential reasons behind tweets' deletion can be hate speech, offensive language, rumors, and/or spam that might violate community guidelines. In such cases, tweets get deleted, and users' accounts could get suspended as well.^1^^,^ ^2^

Bhattacharya and Ganguly (2016) stated that around 11% of tweets are eventually deleted. Although deleted tweets are not accessible once they are deleted, understanding the potential reasons behind their deletion motivates several researchers to understand and identify the content of regrettable tweets or tweets of suspended accounts (Zhou et al., 2016; Gazizullina and Mazzara, 2019). The importance of understanding the content of deleted tweets is the extraction of meaningful data of harmful content, and detecting and empowering users by sending warnings and suggestions before posts get shared on platforms. Prior studies have investigated detecting deleted tweets, spam accounts, and their behaviors (Alom et al., 2018; Vashistha et al., 2023), and identifying factors for undesirable behavior such as spamming, negative sentiment, hate speech, and misinformation spread from deleted or suspended user accounts (Toraman et al., 2022). Most such studies are limited to the English language or distant supervision approach of labeling and fine-grained analysis.

In this study, we investigate the following research questions:*RQ1:* What are the potential reasons (e.g., hate speech and offensive language) behind tweets' deletion?*RQ2:* Are deleted tweets a good way to collect different kinds of harmful content without imposing biases (ex: vs. using keywords)?*RQ3:* How does Twitter deal with users who post disinformative content?*RQ4:* Can we detect the potentiality of deletion of tweets and the corresponding reasons before they are posted?

**Figure 1:**
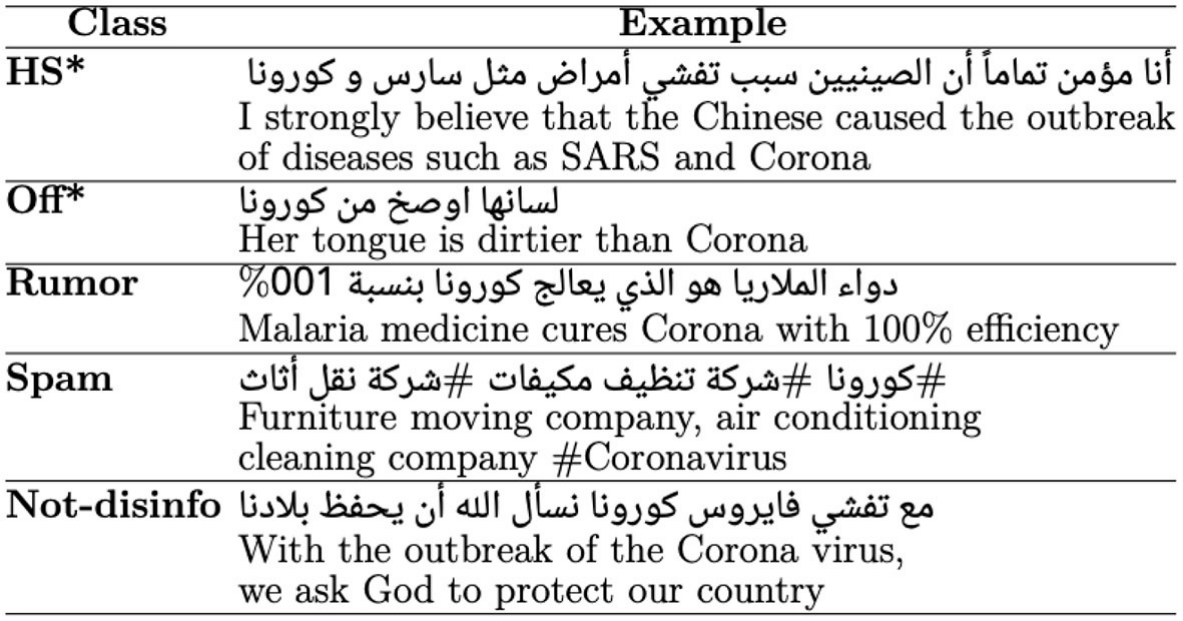
Examples of disinformative and not-disinformative tweets. Not-disinfo, Not disinformative; HS, Hate speech; Off, Offensive. *WARNING: Some examples have offensive language and hate speech, which may be disturbing to the reader.

To address these questions, we collected 40K deleted and non-deleted *Arabic* tweets, and randomly selected a sample of 20K deleted and 2K non-deleted tweets. We then manually labeled them with fine-grained disinformative categories as shown in Figure 1 (see Section 3). Using the labeled dataset, we trained models using classical algorithms (i.e., SVM and RF) and transformer models that can detect the potentiality of tweets getting deleted and the reasons for deletion. From our manual analysis, we found disinformative tweets with a proportion of 20 and 7% in deleted and non-deleted tweets, respectively. This clearly answers the question of deleted tweets being a good way to collect different kinds of harmful content, which can help in developing datasets and models to address disinformative content identification.

Our contributions and findings are summarized as the following:

- We curate and develop a manually labeled dataset consisting of binary labels (deleted vs. non-deleted) and fine-grained disinformative categories. Our data collection method is generic and can be potentially applied to other languages and topics.
- Our proposed “*detection and reasoning of deleted tweets”* approach can empower users by providing feedback before tweets are posted, which can also serve as a prevention mechanism while consciously and unconsciously producing and sharing disinformative posts.
- We report insightful characteristics of deleted tweets' users by extracting their current activity status.
- Our findings demonstrate that deleted tweets contain more disinformation than non-deleted ones.

The paper contains the following sections: Section 2 presents an overview of the related literature, while Sections 3 and 4 discuss the dataset used and provide an analysis of the dataset, respectively. Section 5 details the experiments conducted and the corresponding results, while Section 6 lists some of the limitations in the proposed study, and finally, the paper is concluded in Section 7.

## 2. Related work

Many research investigations have been conducted in the field of regretted and deleted social media data. However, what the literature lacks is the value deleted tweets could have if used as a source of data for essential NLP tasks such as disinformation detection.

### 2.1. Disinformative content detection on social media

Many researchers have explored automatic detection of disinformation on social media. For instance, Demilie and Salau (2022) explored the detection of fake news and hate speech in Ethiopian social media, in which they found that applying a mixture of deep learning and machine learning techniques within the system seemed to be the most effective at identifying disinformation on Ethiopian social media.

In the context of Arabic social media, numerous researchers have employed different approaches to disinformation detection. For instance, Boulouard et al. (2022) investigated disinformation detection, particularly hate speech and offensive content detection, on Arabic social media. By applying transfer learning techniques, they found that BERT (Devlin et al., 2019) and AraBERT (Antoun et al., 2020) performed the best at an accuracy of 98 and 96%, respectively. Mohaouchane et al. (2019) explored the detection of offensive content on Arabic social media through the use of deep learning. By exploring different types of neural networks and training them using AraVec (Soliman et al., 2017) embeddings of the Arabic social media training data, they found that a CNN model achieves the highest accuracy score of 87.84%, while a combined CNN-LSTM model achieves the highest recall at 83.46%.

Such interest in the topic leaves more room for finding ways to extract new data to be used and shared within the community to further improve the current literature, which is where deleted tweets could fill such a gap.

### 2.2. Analysis and detection of deleted tweets

In studies concerning deleted tweets, Almuhimedi et al. (2013) began with a set of 292K unique Twitter users. From these, they extracted all public tweets, retweets, and replies to these posts, along with all relevant metadata for each tweet. Using the API, the authors could determine if a tweet had been deleted, as “a deletion notice was sent via the API containing identifiers for both the user and the specific tweet” (Almuhimedi et al., 2013). This process resulted in a collection of 67.2M tweets, of which 65.6M remained undeleted and 1.6M were deleted. Upon further examination, the authors found that typos and spam, which they considered “superficial” reasons for deletion, accounted for 17 and 1% of the deleted tweets, respectively. Overall, the authors' analysis identified some common reasons for tweets' deletion. They also found that deleted and undeleted tweets share many common characteristics including the topics discussed within those tweets. Taking it a step further, Bhattacharya and Ganguly (2016) investigated the personality of users on Twitter by comparing users who deleted their tweets with the ones who did not. They started by randomly selecting 250K Twitter users and collected their corresponding tweets throughout August 2015, as well as their corresponding deletion statuses.

Current literature suggests that deleted tweets are more likely to have aggressive and negative emotions. Torres-Lugo et al. (2022) analyze “abusive” deletion behavior on Twitter. Using the Compliance Firehose Stream provided by Twitter, they extracted users who had more than 10 deletions over a 1-month period, which amounted to approximately 11 million users. They analyzed abusive deletion behavior by extracting deletion volume, as well as the frequency and lifespan of deleted tweets. They found that “abusive” deleters tend to make use of this feature in order to manipulate the current 2,400 tweets a day limit set by Twitter. Other abusive deleters tend to continuously like and dislike a tweet in order to coordinate which tweets are to be more noticed by other users before deleting them. Lee (2023), on the other hand, analyzed the motivations behind posting on Twitter and how that influences the likelihood of regretting a post and deleting it. The author observed that one of the biggest motivations behind using Twitter is to share opinions, and in fact, users tend to delete their posts to avoid receiving any judgments or hostility from their followers regarding any of the opinions that they might express through social media posts.

Other researchers analyzed features and characteristics of deleted tweets with the goal of training models to predict the likelihood of deletion based on a number of features. Potash et al. (2016) made use of topic modeling and word embeddings to predict whether a tweet is likely to be deleted or not, focusing on spam content. Using features such as tweet length, # of links, ratio of upper-case text, hashtags, etc., they trained multiple classifiers and tested them on a variety of datasets, resulting in a precision of approximately 81%. Similarly, Bagdouri and Oard (2015) investigated the likelihood of a tweet getting deleted within 24 h of the time it was posted. By analyzing the features of both the deleted tweet and the features of the corresponding users, they determined that tweets' features play a significant role in determining the likelihood of deletion. They specifically found that the device used to post the tweet is an important factor in determining deletion's potentiality. For instance, tweets posted using smartphones were more likely to get deleted than those posted via computers. Furthermore, Gazizullina and Mazzara (2019) utilized the Recurrent Neural Networks (RNN) to predict a tweet's likelihood of deletion using features about the text itself, as well as the metadata of tweets and users. Using post-processed word embeddings, they proposed a “Slingshot Net Model” which was evaluated at an F-1 score of 0.755.

While a significant amount of research has delved into the characteristics of deleted tweets, to the best of our knowledge, little attention has been paid to the role of disinformation in the deletion of tweets, particularly in the Arabic context. Thus, we aim to build upon existing literature by investigating the attributes of Arabic deleted tweets and pinpointing various forms of disinformation they might contain. Considering our experiments rely on our proposed dataset, a direct end-to-end comparison with prior literature is not feasible. Nonetheless, our methods for data collection, annotation, and experimentation are poised to benefit the research community. Even though the results are not directly comparable, the performances of the proposed transformer-based models outperform those reported by Gazizullina and Mazzara (2019) and Mohaouchane et al. (2019).

## 3. Dataset

### 3.1. Data collection

We used Twarc package^3^ to collect Arabic tweets in February and March 2020 having the word Corona in Arabic The collection includes 18.8M tweets from which we took a random sample of 100K and checked their existence on Twitter in June 2022. We found that 64K tweets were still active, and 36K tweets were unavailable. The reasons for tweets' unavailability might be due to *(i)* users deleted tweets, *(ii)* deleted accounts, *(iii)* suspended accounts, or *(iv)* accounts became private. Note that accounts' deletion and suspension could also happen due to content violation of Twitter's policies.

We selected a sample of tweets for the annotation in two phases, deleted and non-deleted tweets, respectively. In the *first phase*, a random sample of 20K deleted tweets was selected for the manual annotation with fine-grained disinformative categories (see the following section). In the *second phase*, we selected another 20K non-deleted tweets. From this set, we manually annotated a random sample of only 2K tweets with fine-grained disinformative categories. The reason for the two phases of annotation from both deleted vs. non-deleted tweets was to see if there were similar proportions of disinformative categories in both sets. This also resulted in having an equal sample of 40K deleted and non-deleted tweets which we used for the classification.

### 3.2. Annotation

For the annotation, we selected major harmful categories (i.e., hate speech and offensive) discussed in Alam et al. (2022); Sharma et al. (2022). Additionally, we selected rumor and spam categories as such content is posted on social media. Note that the intention behind rumors is not always harmful; however, due to the spread of false rumors on social media, they can turn out to be harmful (Jung et al., 2020). According to Twitter policies^4^, these types of content are considered as platform manipulation content (“bulk, aggressive, or deceptive activity that misleads others and/or disrupts their experience”).

We use the term “disinformative” to refer to *hate speech (HS), offensive (Off), rumor, and spam*. It is worth mentioning that not all categories directly fall under disinformation; however, we use this term to distinguish such categories from non-disinformative ones.

As for the annotation instructions, we follow the definition of these categories discussed in prior studies: hate speech (Zampieri et al., 2020), offensive (Alam et al., 2022; Sharma et al., 2022), rumors (Jung et al., 2020), and spam (Mubarak et al., 2020; Rao et al., 2021). We asked annotators to select the *non-disinformative* label if a tweet cannot be labeled as any of the disinformative categories we used in this study.

The annotation process consists of several iterations of training by an expert annotator, followed by a final annotation. Given that tweets are in Arabic, we selected a fluent Arabic annotator who is familiar with many Arabic dialects, with an educational qualification of a master's degree.

As mentioned earlier, in the *first phase* we selected and manually annotated 20K deleted tweets. In the *second phase*, we manually annotated 2K non-deleted tweets, and the rest of the 18K tweets of this phase are weakly labeled as *non-disinformative*.

To ensure the quality of the annotations, two annotators initially annotated a randomly selected sample of 500 tweets during the first phase (comprising 250 non-disinformative and 250 fine-grained disinformative tweets). Afterward, we computed the annotation agreement (as detailed in the next section). Considering the expense of the annotation process, we did not assign more than one annotator for the subsequent tweet annotations.

### 3.3. Annotation agreement

We assessed the quality of the annotations by computing inter-annotator agreement from the annotation of three annotators. We computed Fleiss κ and average observed agreement (AoE) (Fleiss et al., 2013) which resulted in an agreement of 0.75 and 0.84, respectively. Based on the values, we reached *substantial* agreement in the κ measurement and *perfect* agreement in the AoE measurement.^5^

### 3.4. Statistics

In Table 1, we report the distribution of annotated tweets (deleted vs. non-deleted tweets). As mentioned earlier, for non-deleted tweets, we manually annotated 2K tweets, and the rest of them are weakly labeled as non-disinformative. From the table (phase 1 column), we observe that the distribution of disinformative tweets is relatively low compared to non-disinformative tweets, which are 19.7 and 80.3%, respectively. From the given sample, 2K non-deleted manual annotated tweets (3rd column), we observe that the distribution between disinformative vs. non-disinformative tweets is 7.3 and 92.7%, respectively. Such a distribution clearly shows us that the distribution of disinformative tweets is more in deleted tweets than non-deleted tweets. This answers the first two questions (RQ1 and RQ2).

**Table 1:** Distribution of annotated tweets.

**Class label**	**Phase 1 deleted**	**Phase 2 non-deleted (2K sample)**	**Phase 2 non-deleted**
Not-disinfo	16,066	1,854	19,854
HS	2,180	58	58
Off	735	47	47
Rumor	252	29	29
Spam	767	12	12
Total	20,000	2,000	20,000

In the 4th column, we show the total number of tweets manually and weakly labeled from non-deleted tweets.

## 4. Analysis

We present an in-depth analysis of the deleted tweets dataset to gain a better understanding of the topics and entities being tweeted about, in relation to COVID-19, and the users who authored those tweets. This includes identifying *(i)* most common rumors discussed about COVID-19 within this dataset; *(ii)* the most common hate-speech targets within the dataset; *(iii)* the current activity status of the users to analyze the potential role that could have been played in the deletion of their tweets; and other metadata such as the distribution of different attributes (e.g., hashtags and user mentions), retweet, and follower counts.

### 4.1. Rumors

When doing the manual annotation, we kept track of the frequent rumors based on the semantic meaning.^6^ The most common rumors were regarding finding potential cures and/or medication to battle COVID-19, while other rumors were related to conspiracies regarding the long-term effects of COVID-19 on humans, as well as potential preventative measures to minimize the spread of the virus. In Table 2, we list the most frequent rumors shared by users included within the dataset in descending order of frequency.

**Table 2:** Most frequent rumors.

**Examples**
1. A number of drugs, including Malaria, Influenza, and AIDS drugs help coronavirus patients improve.
2. Coronavirus is an American invention.
3. Coronavirus is a biological warfare weapon, and many people and novels predicted the virus ahead of time.
4. Coronavirus damages organs of the human body such as the brain and genitals as it causes male infertility.
5. Having certain foods such as tea, maamoul, and gum prevents the infection of Coronavirus.
6. Religious rituals such as wearing niqab, burning incense, being Muslim, and ablution prevents the infection.

Translated forms of Arabic tweets.

### 4.2. Hate speech targets

**Figure 2:**
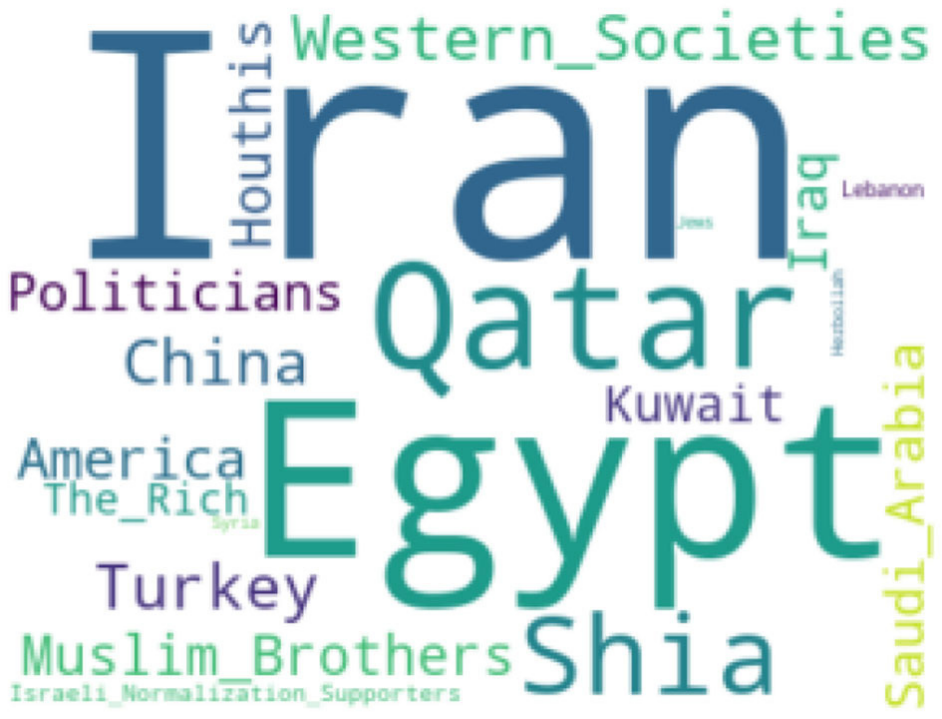
Word cloud for most frequent hate-speech targeted topics/entities.

We wanted to understand if hate speech was targeted toward any entities, countries, or organizations. During the manual annotation, we identified targets to which hate speech has been targeted. We then identified the most frequent entities mentioned throughout tweets classified as hate speech. Countries, political parties, and religion seem to be the most common entities found in tweets that include hate speech words/phrases. In Figure 2, we report the most frequent hate speech targets.

### 4.3. User status

We wanted to understand if there was any association between disinformative categories and current Twitter users' status. The goal was to understand whether the current status of a given account was a major factor in deleting tweets. Also, if the account gets deleted or suspended, tweets of such an account get deleted as well. Using the information provided by Twitter API, we determined the current user status of all unique users who posted at least one disinformative tweet. In total, there were 3,677 unique users who posted at least one disinformative tweet. Each of the unique users was classified under one of four categories: suspended (removed by Twitter), deleted (initiated by the user), active-private (user is active but private, blocking public access to any of their tweets), and active-public (user is active, and their tweets are publicly available).

**Figure 3:**
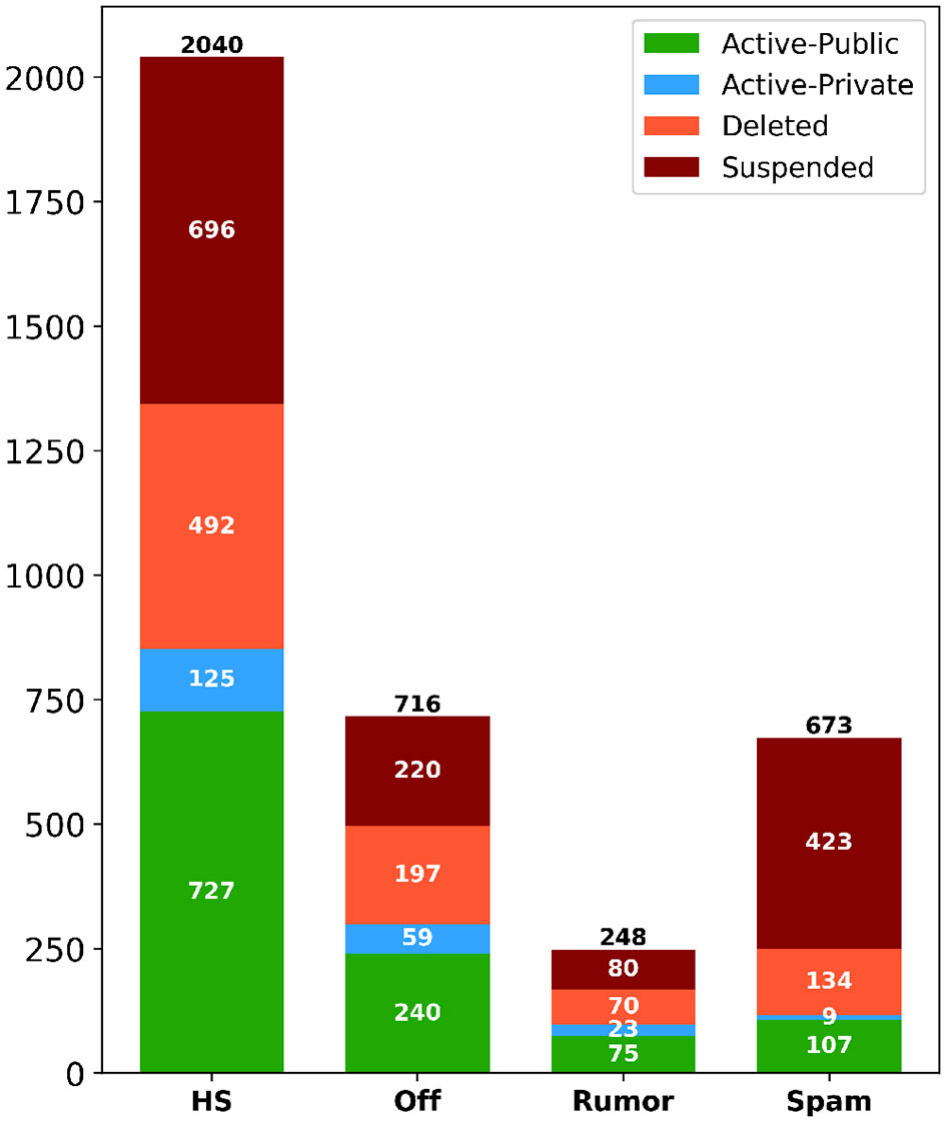
Distribution of users' account status corresponding to each disinformative category. This status is based on the time of our analysis period (August 2022).

In Figure 3, we present the number of users' accounts for each disinformative category. From the figure, we observe that the distribution of hate speech is higher than in other categories. An interesting point to note is that almost 40% (1,419) of all users, with at least one disinformative post, were suspended by Twitter. Out of those users, Twitter was very efficient at identifying and disabling spam users, as it could suspend 423 accounts of users who shared at least one spam tweet, which amounts to more than 62% of accounts that posted any spam content. In respect of hate speech posters, Twitter identified and suspended over 34% (696) of them. For, the other accounts, approximately 24% (893) of them were deleted by the users themselves, while 6% (216) of them are currently active but are set to private, and the remaining 33% (1,224) are still active and public. This analysis answers RQ3, as it shows that Twitter is able to identify some users who post disinformative content, and ultimately suspend the whole account.

As a result, user status is an important factor to take into consideration when analyzing and characterizing the deletion of tweets, as it could be due to their corresponding accounts not existing anymore, either as a result of Twitter suspension, user deactivation, or the user setting the account to private.

### 4.4. Other metadata

In Table 3, we report the distributions of some attributes in the non-deleted, deleted, and associated disinformative tweets. There are minor differences between the non-deleted and disinformative tweets. However, the subset of the deleted tweets that are labeled as disinformative has different distributions. For example, disinformative tweets have double as many URLs, as well as more replies than the other sets, and they are less likely to be retweeted by one-seventh (12% vs. 77% or 82%).

**Table 3:** Percentages of tweets having different attributes.

**Attributes**	**Non-deleted (%)**	**Deleted (%)**	**Disinformative (%)**
Hashtags	57	55	**63**
URLs	29	25	**51**
User mentions	82	**87**	24
Replies	05	05	**09**
Retweets	77	**82**	12

From this dataset, we also observe that the percentage of hate speech is higher than in other categories, which might be due to the topic of interest, i.e., COVID-19. Similar findings are reported in Mubarak and Hassan (2020), which suggest that tweets about COVID-19 were found to have a higher percentage of hate speech (7%) as it is a polarized topic, e.g., attacking some countries for spreading the virus. This is typically different from random collections of Arabic tweets. Mubarak et al. (2021) reported that the percentage of offensive language in random collections is between 1 and 2%, and the hate speech ratio is even less.

We hypothesize that many of the deleted tweets contain more harmful content than normal (e.g., 10.9% hate speech and 3.8% spam), and Twitter deleted them as they violated its community standards or they were deleted by the users themselves as they regretted posting some tweets because they contain offensiveness or rumors. This also answers our first two research questions.

## 5. Experiments and results

**Figure 4:**
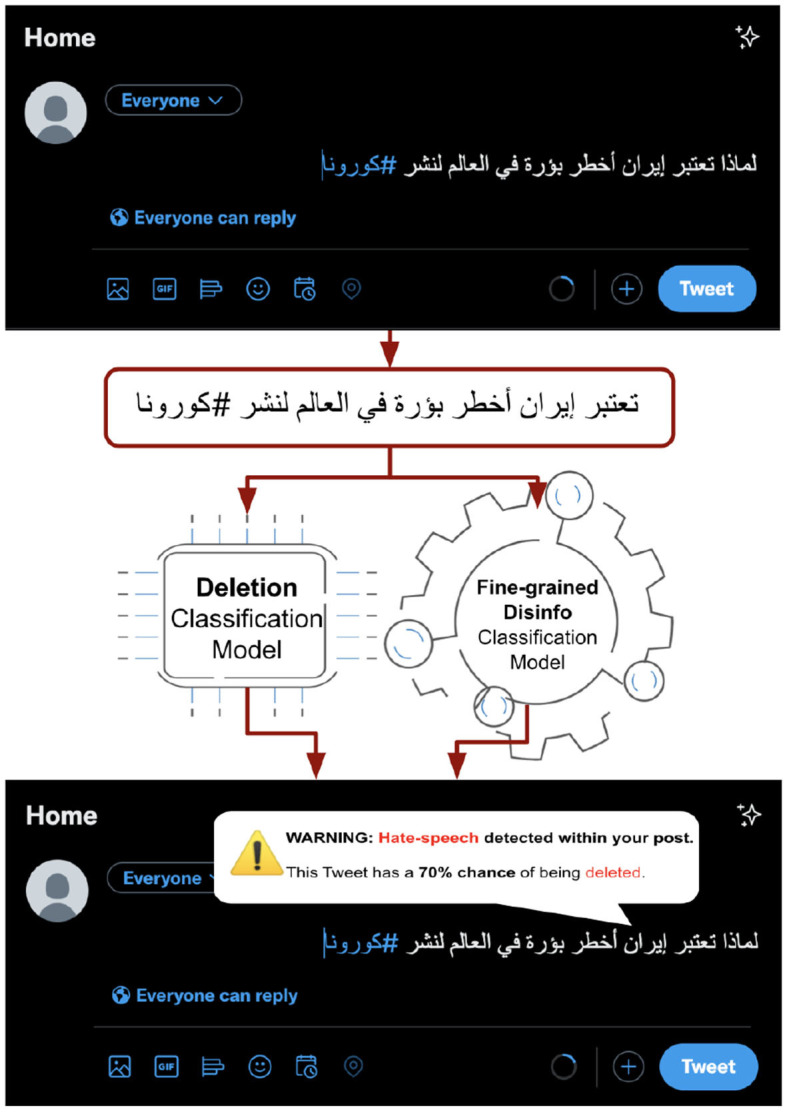
A pipeline of our proposed system to detect and warn users while posting—what can happen and why. Translation (HS*): *Why is Iran considered the most dangerous spot in the world for spreading Corona?*

In Figure 4, we present our proposed pipeline of post-deletion detection with reasons for posting on social media. While posting the tweet detection model can detect whether a tweet will be deleted, and the fine-grained disinformation model can detect whether it is one of the disinformation categories (e.g., in this case, hate speech). Our goal is to empower users while posting and/or sharing content and reduce the spread of misleading and harmful content. In the following sections, we describe the details of the proposed models and results.

### 5.1. Experiment settings

We have conducted different classification experiments with a focus on detecting whether a tweet can be deleted before posting, and what could be the possible reasons. We train three different classifiers as follows: *(i)* a binary classifier to detect whether a tweet will be deleted using the labels *deleted* vs. non-deleted tweets, which consists of 40K tweets; *(ii)* a binary classifier to detect whether a tweet is disinformative vs. non-disinformative, *(iii)* a multiclass classifier to detect fine-grained disinformative categories. For the latter two classifiers, we used manually labeled 22K tweets. Note that we have not used all 40K for the latter two sets of experiments given that they are weakly labeled (18K considered as non-disinformative) tweets. This could be a part of our future study.

### 5.2. Data splits and preprocessing

To conduct experiments, we split our dataset into three subsets with a 70-10-20 setting for train, dev, and test sets, respectively. The class distributions within each subset are shown in Table 4. The second set (ii) of data split in the table is a subset of the first set, whereas the third set (iii) is only fine-grained *disinformation* categories of the second set (ii).

**Table 4:** Distribution of the dataset for different experimental settings for train, dev, and test sets.

**Class label**	**Train**	**Dev**	**Test**	**Total**
**(i) Binary: Deleted vs. Non-deleted**				
Deleted	14,012	2,020	3,968	20,000
Not-deleted	13,988	1,980	4,032	20,000
Total	28,000	4,000	8,000	40,000
**(ii) Binary: Disinfo vs. Non-disinfo**				
Disinformation	2,879	394	807	4,080
Not-Disinfo	12,521	1,806	3,593	17,920
Total	15,400	2,200	**4,400**	**22,000**
**(iii) Multiclass: Fine-grained disinfo labels**				
HS	1,563	227	448	2,238
Off	554	83	161	798
Rumor	189	31	61	281
Spam	550	67	146	763
Total	2,856	408	816	4,080

#### 5.2.1. Preprocessing

Given that social media texts are usually noisy, before any classification experiments, we applied preprocessing to the dataset. The preprocessing includes the removal of hash symbols and non-alphanumeric symbols, URL replacement with a “URL” token, and username replacement with a “USER” token.

### 5.3. Models

We experimented with binary and multiclass settings both classical and deep learning algorithms discussed below. The classical models include *(i)* Random Forest (RF) (Breiman, 2001), and *(ii)* Support Vector Machines (SVM) (Platt, 1998), which was most widely reported in the literature. The other reason to choose such algorithms is that they are computationally efficient and useful in many production systems.

Given that large-scale pre-trained Transformer models have achieved state-of-the-art performance for several NLP tasks, therefore, as deep learning algorithms, we used deep contextualized text representations based on such pre-trained transformer models. We used AraBERT (Antoun et al., 2020) and multilingual transformers such as XLM-R (Conneau et al., 2019). Our motivation for choosing AraBERT and XLM-R was influenced by our prior work on the large-scale classification of COVID-19-related tweets, which involved multiple categories of disinformation (Alam et al., 2021). The study reports that both models perform comparably on the Arabic dataset. For Transformer models, we used the Transformer toolkit (Wolf et al., 2019). We fine-tuned each model using the default settings for 10 epochs as described in Devlin et al. (2019). We performed 10 reruns for each experiment using different random seeds and selected the model that performed best on the development set. More details of the experimental parameters are discussed below.

### 5.4. Details of the experiments

For experiments using SVM and RF, we employed standard parameter settings. We transformed the text into a tf-idf representation before inputting it to the SVM and RF models. For the experiments with transformer models, we adhered to the following hyper-parameters during the fine-tuning process. Additionally, we have released all our scripts for reproducibility.

- Batch size: 8;
- Learning rate (Adam): 2e-5;
- Number of epochs: 10;
- Max seq length: 128.

**Models and parameters:**

- **AraBERT** (bert-base-arabert):L = 12, H = 768, A = 12, total parameters: 110M; where *L* is the number of layers (i.e., Transformer blocks), *H* is the hidden size, and *A* is the number of self-attention heads; (110M);
- **XLM-RoBERTa** (xlm-roberta-base): L = 24, H = 1,027, A = 16; the total number of parameters is 355M.

**Computing infrastructure and runtime:** We used a server with NVIDIA Tesla V100-SXM2-32 GB GPU, 56 cores, and 256 GB CPU memory.

### 5.5. Results and discussion

#### 5.5.1. Results

We report accuracy (Acc), weighted precision (P), recall (R), and F1 scores which take into account the class imbalance that we had in our dataset. We compute the majority as a baseline.

In Table 5, we report the classification experiments of all different settings. From the table, we can see that all models outperform the majority class baseline. Compared to the classical algorithms, SVM outperforms RF in two settings out of three. While comparing monolingual vs. multilingual transformer models, we observe that AraBERT performs well in detecting deleted tweets and XLM-R outperforms well in classifying whether the text of the tweet is disinformative or not. For classifying fine-grained disinformative categories, AraBERT outperforms all other models. Our results clearly answer *RQ4*, in that we can detect the potentiality of deletion of tweets and the corresponding reasons, with reasonable accuracy.

**Table 5:** Classification results for different settings that can detect tweet deletion and possible fine-grained reasons.

**Model**	**Acc**	**P**	**R**	**F1**
**(i) Binary: Deleted vs. Non-deleted**				
Majority	0.496	0.246	0.496	0.329
RF	0.896	0.882	0.896	0.854
SVM	0.852	0.851	0.852	0.850
AraBERT	0.910	0.896	0.910	**0.902**
XLM-R	0.886	0.784	0.886	0.832
**(ii) Binary: Disinfo vs. Non-disinfo**				
Majority	0.817	0.667	0.817	0.734
RF	0.853	0.871	0.853	0.812
SVM	0.837	0.838	0.837	0.837
AraBERT	0.888	0.882	0.888	0.884
XLM-R	0.897	0.894	0.897	**0.895**
**(iii) Multiclass: Fine-grained disinfo labels**				
Majority	0.537	0.288	0.537	0.375
RF	0.696	0.760	0.696	0.622
SVM	0.669	0.677	0.669	0.665
AraBERT	0.755	0.757	0.755	**0.752**
XLM-R	0.762	0.747	0.762	0.745

XLM-R: XLM-RoBERTa. The bold values are for the best performing models (highest f1 scores).

#### 5.5.2. Error analysis

We analyzed all rumors and offensive tweets that are misclassified as hate speech (*n* = 243). We found annotation errors in 18% of the cases, and 5% of the errors are due to sarcasm, negation, or tweets having rumors and hate speech at the same time. In the other cases, the model predicted the label as hate speech as it is the dominant class as shown in statistics in Table 1. By looking into individual class label performance for disinformative categories, we observe that spam and hate speech are the best-performing labels (F1 = 0.940 and F1 = 0.779, respectively). The offensive label is the lowest in performance (F1 = 0.513), which is due to mislabeling as hate speech in many cases.

#### 5.5.3. Comparison with prior studies

As previously discussed, our experimental results are not directly comparable to any existing work due to differences in the dataset and the nature of the problem. In this study, we introduce a novel dataset addressing an issue that has not been explored in the context of deleted Arabic tweets. In terms of experiments, among the three classification settings, XLM-R outperforms other models in two of them. In a related study, Boulouard et al. (2022) demonstrated that BERT and AraBERT achieve better results, reaching an accuracy of 98% in a binary classification setting distinguishing between hate and non-hate labels. In contrast to this work and the aforementioned dataset, our dataset presents a more significant challenge due to its high imbalance between disinfo and non-disinfo labels, as well as its fine-grained categories.

## 6. Limitations

We developed a dataset that consists of tweets extracted from Twitter only. Additionally, we developed models that require further investigation to understand whether models will work on datasets from other social media platforms.

It is important to note that although this exploration looks into the likelihood of tweet deletion based on an annotated dataset, the moderation techniques employed by social media networks such as Twitter require further analysis to be able to gain insight into potential reasons for user suspension and/or tweet deletion.

## 7. Conclusion and recommendation

We presented a large manual annotated dataset that consists of deleted and non-deleted Arabic tweets with fine-grained disinformative categories. We proposed classification models that can help in detecting whether a tweet will be deleted before even being posted and detect the possible reasons for the deletion. We also reported the common characteristics of the users whose tweets were deleted. After trying different settings for training a binary model on identifying deleted vs. non-deleted tweets, a binary model for classifying disinformative vs. non-disinformative tweets, and a multiclass model classifying fine-grained disinfo labels, we find that the best-performing setting for each model achieves an F1 score of 0.902, 0.895, and 0.752, respectively. Such findings suggest that deleted tweets can be used in developing annotated datasets of misinformative and disinformative categories, which make for an interesting advancement in the detection of disinformative content on social media.

Future recommendations include more fine-grained categories that are mostly harmful (e.g., racist) and finding more reasons for tweets' deletion which can empower social media users. In addition, we plan to explore a multitask learning setup that can reduce computational cost and may boost the performance of the model. Also, for future explorations regarding this topic, there needs to be a larger dataset of deleted tweets used that takes into consideration factors such as the account being suspended as opposed to the individual tweet being deleted.

## Data availability statement

The datasets presented in this article are not readily available, since we are working with deleted tweets, we are still exploring the possibility of sharing the tweets while still complying with Twitter guidelines. Requests to access the datasets should be directed to HM, hmubarak@hbku.edu.qa.

## Author contributions

HM: data collection and research idea. SA: data analysis, visualization, and annotation. AN: data annotation. FA: experiments and results. All authors contributed to the article and approved the submitted version.

## Footnotes

^1^ https://help.twitter.com/en/rules-and-policies/twitter-rules

^2^ https://help.twitter.com/en/managing-your-account/suspended-twitter-accounts

^3^ https://github.com/DocNow/twarc

^4^ https://help.twitter.com/en/rules-and-policies/platform-manipulation

^5^Note that, in the Kappa measurement, the values of ranges 0.41-0.60, 0.61-0.80, and 0.81-1 refer to the moderate, substantial, and perfect agreement, respectively (Landis and Koch, 1977).

^6^There are no duplicate tweets; we removed them at the beginning.
